# Gut microbiota: a new target for the prevention and treatment of insomnia using Chinese herbal medicines and their active components

**DOI:** 10.3389/fphar.2025.1572007

**Published:** 2025-05-06

**Authors:** Changmei Wu, Jinjin Dou, Xiaoxue Song, Fang Yang, Xuan Liu, Weipeng Song, Xiwu Zhang

**Affiliations:** ^1^ Graduate School, Heilongjiang University of Chinese Medicine, Harbin, China; ^2^ The Four Hospital of Heilongjiang University of Chinese Medicine, Heilongjiang University of Chinese Medicine, Harbin, China; ^3^ Experimental Teaching and Practical Training Center, Heilongjiang University of Chinese Medicine, Harbin, China; ^4^ First Clinical Medical College, Heilongjiang University of Chinese Medicine, Harbin, China; ^5^ Research Institute of Chinese Medicine, Heilongjiang University of Chinese Medicine, Harbin, China.

**Keywords:** Chinese herbal medicines and their active components, microbiota-gutbrain axis, insomnia, improving sleep quality, Gut Microbiota

## Abstract

The emergence of the microbiota-gut-brain axis has opened new avenues for improving sleep quality. Recent studies have revealed a close relationship between insomnia and the gut microbiome. Chinese herbal medicines and their active components can alter the relative abundance of sleep-related gut microbiota by reversing dysbiosis in the gut microbiome. Improving sleep quality through the regulation of the gut microbiota using herbal medicine and its active components has become a highly promising therapeutic strategy. This article elucidates how the gut microbiota modulates sleep quality via the intricate communication network of the gut-brain axis. It also reviews the latest research on utilizing herbal medicine and its active components to regulate the gut microbiota for enhancing sleep quality. Additionally, it provides insights into the potential of herbal medicine and its active components in improving sleep quality through the modulation of the gut microbiota.

## 1 Introduction

Insomnia is a common sleep disorder characterized mainly by poor sleep quality and insufficient sleep duration. It affects approximately 30% of the global population and is correlated with physical and mental consequences such as cognitive decline, anxiety, chronic fatigue, poor concentration, and memory impairment (Rivero-Segura et al., 2025; Zhang et al., 2025). In recent years, an increasing number of studies have shown a close relationship between the gut microbiota and sleep.

As a bridge for communication between the gut microbiota and the brain, the gut-brain axis is a bidirectional regulatory axis involving interactions between the central nervous system (CNS) and the gastrointestinal tract. It also comprises a neuroendocrine network consisting of the CNS, the autonomic nervous system, the hypothalamic-pituitary-adrenal (HPA) axis, and the enteric nervous system (Arzani et al., 2020; Mayer et al., 2015). Gut microbes can participate in regulating the sleep functions of the brain via the gut-brain axis, either directly or indirectly (Wang et al., 2022; Wu J. et al., 2023). Sleep deprivation (SD) has been shown to reduce the Firmicutes-to-Bacteroidetes (F/B) ratio in rats (Yang et al., 2023). Consuming specific probiotics can effectively improve the diversity of the gut microbiota and enhance sleep quality through microbial metabolic products (Lan et al., 2023; Matsuda et al., 2020; Zhu et al., 2023). Therefore, scientifically intervening with the gut microbiota represents a new and effective target for preventing and treating insomnia.

Chinese herbal medicines and their active components serve as unique ‘prebiotics,’ not only providing energy for the activities of the gut microbiota but also improving the gut microenvironment by modulating the intestinal microbiota, thereby affecting various physiological functions of the body (Álvarez et al., 2022; Cheng X. et al., 2024). They not only offer gentle and sustained regulation of the gut microbiota but also exhibit significant drug-like properties, good pharmacokinetics, and low toxicity when it comes to improving sleep quality (Ranteh et al., 2024). Chinese herbal medicines and their active components hold great potential in improving insomnia through the modulation of the gut microbiota.

This paper summarizes the current state of research on the potential of herbal medicines and their active ingredients to improve sleep quality by modulating the gut microbiota. It also provides an overview of recent studies on the mechanisms by which the gut microbiota can be utilized to prevent and treat insomnia.

## 2 Characteristics of the gut microbiota in patients with insomnia

Insomnia can affect the richness and diversity of the gut microbiota. Compared to the normal group, SD mice exhibit a significant reduction in the abundance of *Akkermansia muciniphila*, *Bacteroides*, and *Faecalibacterium* and a significant increase in the abundance of *Aeromonas* (Gao et al., 2019; Li N. et al., 2023; Moreno-Indias et al., 2015). Compared to the control group, patients with insomnia show significant differences in the abundance of *Collinsella*, *Adlercreutzia*, *Erysipelotrichaceae*, *Clostridiales*, *Pediococcus*, *Bacteroides*, *Staphylococcus*, *Carnobacterium*, *Pseudomonas*, *Odoribacter*, *Bifidobacterium longum*, and *Phascolarctobacterium* (Barone et al., 2024; Zeng et al., 2024). Similarly, patients with obstructive sleep apnea exhibit significant differences in the abundance of *Lactobacillus*, *Ruminococcaceae*, *Proteobacteria*, *Clostridiaceae*, *Oscillospiraceae*, *Klebsiella*, *Desulfovibrionaceae*, *Bacteroides fragilis*, and *Faecalibacterium prausnitzii* (Ko et al., 2019; Valentini et al., 2020).

Whether it is the alteration of strains associated with inflammation, such as *Proteobacteria*, *Clostridiaceae*, *Oscillospiraceae*, and *Klebsiella*, or strains related to gut barrier integrity, such as *Desulfovibrionaceae*, *Bacteroides fragilis*, and *F. prausnitzii*, these changes are significantly correlated with parameters related to sleep quality (Valentini et al., 2020). Insomnia not only reduces the gut’s antioxidant capacity, anti-inflammatory cytokine levels, mucin 2 (MUC2), and tight junction protein expression but also increases the levels of pro-inflammatory cytokines, leading to intestinal mucosal damage and increased barrier permeability (Gao et al., 2019; Li L. et al., 2023). When SD animals’ gut microbiota is transferred into normal mice, the donor animals’ gut microbiota is changed, which results in elevated levels of lipopolysaccharides (LPS) and *Aeromonas*, lowered levels of butyrate and *Lachnospiraceae_NK4A136*, and increased hippocampal microglia activation and neuronal death (Wang X. et al., 2023). However, pretreatment with *Lactiplantibacillus plantarum* 124 or *A. muciniphila* restores the disrupted gut microbiota, reduces oxidative stress, inflammation, and barrier damage in the gut (Li L. et al., 2023), increases acetate and butyrate levels, and prevents synapse loss in microglia-neuron co-cultures stimulated by LPS (Li N. et al., 2023). Thus, disruption of the gut microbiota affects the host’s sleep behavior through mechanisms such as reducing gut barrier integrity and short-chain fatty acid (SCFAs) content and increasing inflammatory mediators that trigger inflammatory responses.

## 3 Gut microbiota involved in the regulation of insomnia mechanisms

### 3.1 Hypothalamic-pituitary-adrenal axis

Insomnia activates and leads to dysfunction of the HPA axis (Luo et al., 2024; Wang Z. et al., 2024). Microbes may influence HPA axis activity through inbound neural signaling, SCFAs, epigenetics, gut barrier integrity, or blood-brain barrier (BBB) permeability (Cao C. et al., 2020; Noemi et al., 2024). Therefore, it is suggested that the dysregulation of gut microbiota caused by insomnia may be related to the activation of the HPA axis (Matenchuk et al., 2020).

**FIGURE 1:**
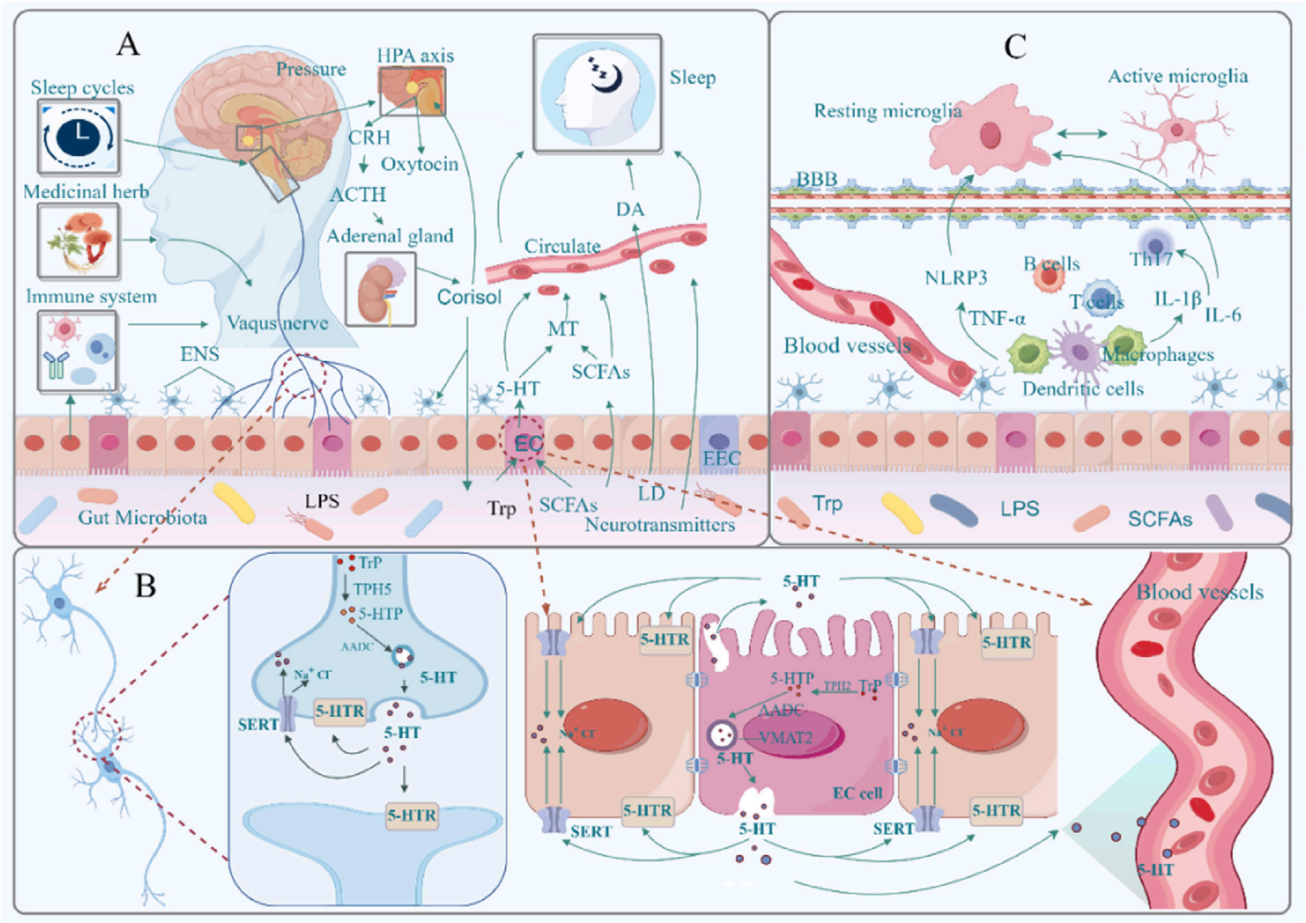
Bidirectional regulatory mechanisms between the gut microbiota and the brain. **(A)**: Microbial endocrine and metabolite pathways, hypothalamic–pituitary–adrenal axis; **(B)**: Microbiota–vagus nerve pathway; **(C)**: Microbiota–immune inflammatory pathway; AANAT, arylalkylamine N-acetyltransferase (a rate-limiting enzyme produced by MT); SCFAs, short-chain fatty acids; 5-HT, 5-hydroxytryptamine; MT, melatonin; DA, dopamine; HPA, hypothalamus-pituitary-adrenal axis; CRH, adrenocorticotropic hormone-releasing hormone; ACTH, adrenocorticotropic hormone; EC, enterochromaffin cells; EEC, intestinal endocrine cell; Trp, tryptophan; 5-HTR, 5-hydroxytryptamine receptor; 5-HTP, 5-hydroxytryptophan; TPH2, tryptophan hydroxylase 2; TPH5, tryptophan hydroxylase 5; SERT, 5-hydroxytryptamine transporter; VMAT2, vesicular monoamine transporter 2; MAO, monoamine oxidase; BBB, blood-brain barrier; LPS, lipopolysaccharide; TNF-α, tumor necrosis factor α; NLRP3, NOD-like receptor thermoprotein domain protein 3; IL-6, interleukin-6; IL-1β, interleukin-1β; Th17, helper T cell 17. Materials provided by Fig Draw (www.fgdraw.com).

Disruption of the HPA axis is associated with an increase in pathogenic bacteria (including *Enterobacteriaceae*, *Streptococcaceae*, and *Veillonellaceae*) and a decrease in beneficial bacteria (including *Bifidobacterium* and *Lachnospiraceae*) (Jahnke et al., 2021). Certain specific gut microbiota can inhibit HPA axis activation and mediate related brain functions. *Escherichia coli* and *Enterococcus faecalis* can lower cortisol levels in the serum of mice, thereby alleviating stress and depressive-anxiety behaviors (Luo et al., 2018; Wu et al., 2021). Analysis indicates that oxymesterone in feces can mediate the negative effect of cortisol on *Parabacteroides*, while 3-(2,4-cyclopentadien-1-yl)-5α-androstan-17β-ol can mediate the negative effect of cortisol on *Aerococcus*; mevinolinic acid can also mediate the negative effect of *Aerococcus* on cortisol (Wang J. et al., 2023). This suggests that signaling between the gut microbiota and the HPA axis is bidirectional (Figure 1).

### 3.2 Microbial endocrinological products and metabolites

#### 3.2.1 Melatonin

As a “dark hormone,” melatonin (MT) is crucial for maintaining the body’s normal sleep-wake cycle. Under normal conditions, less than 5% of tryptophan (Trp) in the human body is used for the synthesis of 5-hydroxytryptamine (5-HT) and MT, while the remaining 95% is metabolized through the kynurenine (Kyn) pathway in the liver (Muneer, 2020). Long-term stress can disrupt the balance of Kyn metabolism and endocrine function along the gut-brain axis, leading to dysbiosis in the gut microbiota (Deng et al., 2021). This dysbiosis further stimulates the Kyn pathway, diverting Trp that should be directed towards MT synthesis, thus reducing circulating MT levels (Yan et al., 2023).

In addition to restoring the circadian rhythm equilibrium and regulating the composition of the gut microbiota, exogenous MT supplementation has been shown to raise SCFAs levels and modify communication between the gut microbiota and the brain (Gao et al., 2021; Li et al., 2023a; Li et al., 2023b). Supplementing with MT, for instance, can improve cognitive impairment caused by SD by reducing hippocampal inflammation and neuronal apoptosis, increasing the relative abundance of *Lachnospiraceae_NK4A136* and butyrate levels, decreasing the relative abundance of *Aeromonas* and LPS levels, and regulating the TLR4/NF-κB and MCT1/HDAC3 signaling pathways (Wang X. et al., 2023).

Certain microorganisms in the gut can promote the synthesis of MT. Studies have shown that *Lactobacillus reuteri* and *E. coli* can activate the TLR2/4/MyD88/NF-κB signaling pathway, promoting the expression of arylalkylamine N-acetyltransferase (AANAT), which in turn drives the synthesis of MT (Liu et al., 2024). Microbial metabolites such as SCFAs can also mediate the transcription of AANAT by increasing 5-HT levels and promoting the phosphorylation of cAMP response element-binding protein (p-CREB), thereby facilitating MT synthesis (Song et al., 2021) (Figure 1). Notably, these MT molecules originating from the gut may act on MT1 or MT2 receptors in the brain through the circulatory system, thereby regulating sleep behavior in the organism (Li B. et al., 2023).

#### 3.2.2 γ-Aminobutyric acid

γ-Aminobutyric acid (GABA) is a well-known sleep-promoting amino acid. In the intestinal mucosa, there exists a line of entero-neuroendocrine cells: STC-1 and STC-2, which can express mRNA for GABAA receptors and their receptor subtypes (α1, α3, α5, β1, β3, and δ) (Noguchi et al., 2024). Besides the ability of gastrointestinal neuroendocrine cells to synthesize, store, and secrete GABA, certain members of the gut microbiota can also produce GABA.

*Lactic acid bacteria* and *bifidobacteria* are key members in the production of GABA. *Bifidobacteria* colonizing the mucus layer of the gut possess enzymatic mechanisms capable of converting glutamate (Glu), glutamine (Gln), and succinate into GABA. GABA derived from the gut can maintain the intestinal barrier and its function by stimulating Ca^2+^ signaling, MUC2 release, and proliferation of intestinal stem cells (Engevik et al., 2019; Luck et al., 2021; Zhou et al., 2023). *Limosilactobacillus fermentum* L18 can secrete high levels of GABA, enhancing the gut barrier by increasing the concentration of connexins and the abundance of beneficial gut bacteria (Kaur et al., 2023). *Lactobacillus rhamnosus* GG, in addition to protecting the gut barrier, can also enhance brain-derived neurotrophic factor (BDNF) and GABA receptor levels in the hippocampus and amygdala by modulating the gut-brain axis (Zhou et al., 2022). Additionally, studies have shown that the abundance of *Bacteroides* is positively correlated with the expression of GABA receptor proteins (Bedu-Ferrari et al., 2024; Duan H. et al., 2023).

Relevant research has demonstrated that high doses of GABA produced by *Lactobacillus brevis* fermentation can raise the relative abundance of good bacteria in the gut and the levels of SCFAs, which in turn can upregulate the GABAergic and 5-HTergic neurotransmitter mRNA and protein expression levels. This will result in a significant increase in theta (θ) and delta (δ) waves and non-rapid eye movement (NREM) sleep (Jeong et al., 2021; Yu et al., 2020). By altering the distribution of gut microbiota and upregulating the production of GABAA receptor proteins in gut tissue, the formula for spleen deficiency and liver qi stagnation enhances the quality of sleep (Duan H. et al., 2023). This has therapeutic benefits for depression and insomnia. It is clear that gut-derived GABA can act as a conduit for information between the brain and the microbiota, helping to regulate sleep behavior via the gut-brain axis and enhancing the organism’s quality of sleep.

Notably, the BBB has very poor permeability to GABA, and whether GABA can cross the BBB to act on receptors derived from the central nervous system remains questionable. However, there are large numbers of GABA receptors present in the enteroendocrine cell lines of the gut (Noguchi et al., 2024). Therefore, it is suggested that the impact of exogenous and gut-derived GABA on brain function may be achieved directly through the enteric nervous system (Tri et al., 2023).

#### 3.2.3 Serotonin

As a key neurotransmitter in the gut-brain axis, 5-hydroxytryptamine (5-HT, serotonin) serves as an important mediator allowing communication between gut microbiota and the brain. Approximately 95% of 5-HT in the body originates from the gastrointestinal (GI) tract (Liu et al., 2021). Within the GI tract, sources of 5-HT include the conversion of Trp to 5-hydroxytryptophan (5-HTP) via tryptophan hydroxylase (TPH) 1 in enterochromaffin (EC) cells and TPH2 in neuronal cells, followed by the transformation into 5-HT through aromatic L-amino acid decarboxylase (AADC) (Layunta et al., 2021); or the induction of 5-HT secretion from EC cells triggered by calcium (Ca^2+^) influx via noncanonical signaling pathways initiated by interleukin 33 (IL-33) (Chen et al., 2021); and SCFAs promote synthesis by regulating the serotonin transporter (SERT), 5-HT receptors, and inhibiting the conversion of Trp to kynurenine (Buey et al., 2023; Xiao W. et al., 2022).

The variety of the gut microbiota affects 5-HT levels, which in turn impacts the organism’s sleep patterns. Supplementing with Trp and 5-HTP has been shown to improve the diversity of the gut microbiota, increase the amount of SCFAs in the gut, and raise blood levels of 5-HT, all of which improve the quality of sleep for both elderly people and neonates (Chojnacki et al., 2023; Sutanto et al., 2024). After depleting the microbiota with antibiotics, the expression levels of 5-HT decrease, leading to an increase in the onset frequency of REM sleep, frequent transitions between NREM and REM sleep, and ultimately having a negative impact on sleep (Ogawa et al., 2020). For example, *Ganoderma lucidum* can regulate the gut microbiota and 5-HT-related pathways, increasing serum levels of 5-HT and GABA while decreasing HPA axis hormone levels, effectively alleviating insomnia behavior in anxious mice (J. H. Huang et al., 2022; Yao et al., 2021). Clearly, microbiota can modulate sleep behavior in the brain by influencing the synthesis and release of 5-HT in the gut (Figure 1).

#### 3.2.4 Short-chain fatty acids

SCFAs are the primary metabolites produced by gut bacteria and are believed to be neuromodulatory substances (Gunawan et al., 2024). SCFAs are essential for the gut microbiota’s control of sleep. Through immunological, neurological, and endocrine pathways, the microbiota forms close connections with brain sleep processes; the “gut-brain axis” serves as a link between the two, and SCFAs function as messengers (Figure 1).

Insomnia has been shown to decrease the number of microorganisms that generate SCFAs, such as *Faecalibacterium*, *Roseburia*, and *Ruminococcaceae*, which in turn lowers the amount of SCFAs in the gut (Shimizu et al., 2023; Wu et al., 2022). Elevated gut SCFAs levels have been shown to enhance the quality of sleep (Heath et al., 2020). Relevant studies have demonstrated that elevated SCFAs levels can affect brain development by crossing the blood-brain barrier and regulating the production of 5-HT and DA in the brain (Wang et al., 2020), in addition to helping the brain regulate sleep behavior by increasing 5-HT and MT levels in the gut and inhibiting HPA axis hormone levels (Li B. et al., 2023; Li et al., 2024; Ogawa et al., 2020; Wang J. et al., 2024).

Moreover, SCFAs have the ability to influence ILC3, T cells, and B cells in the gut, which helps to control the intestinal barrier’s immunological balance (Kim, 2021). In addition to counteracting age-related microbiome dysbiosis by attenuating the expression of pro-inflammatory cytokines in microglia, SCFAs also activate colonic NLRP6 inflammasomes, improving damage to the intestinal epithelial barrier and reducing neuroinflammation and neuronal loss in the hippocampus (Vailati-Riboni et al., 2022; Yu et al., 2021). The neural system and certain immunological signaling molecules in the brain can interact to help regulate sleep (Piber et al., 2022).

SCFAs produced by microbiota can affect the activity of the HPA axis. By significantly increasing SCFAs content, it is possible to restore the expression of tight junction genes (OCLN and TJP2) in the hypothalamus and hippocampus and significantly modulate the expression of corticotropin-releasing hormone receptor genes CRF1 and CRF2, thereby alleviating depression and anxiety induced by SD (Chung et al., 2023). Bifidobacterium breve 207-1 can improve sleep quality by significantly increasing SCFAs and GABA levels and overall suppressing HPA axis-related hormones (Li et al., 2024). Acetate, butyrate, and valerate show a negative correlation with HPA-axis-related hormone levels; propionate shows the opposite pattern (Wang J. et al., 2024).

Thus, SCFAs produced by microbiota can profoundly influence the nervous system by improving the gut barrier and the gut immune environment. They achieve the goal of improving insomnia through mechanisms such as increasing the diversity and levels of SCFAs in the gut, stimulating the secretion of sleep-related cytokines, and inhibiting inflammatory responses.

#### 3.2.5 Dopamine

Dopamine (DA), a powerful neurotransmitter that promotes alertness, is essential for controlling sleep-wake cycles (Wu et al., 2024). DA strengthens the gastrointestinal barrier and defense mechanisms in the gastrointestinal tract by stimulating the secretion of distal colon mucus via D5 receptors and improving the secretion of gastrointestinal bicarbonate through D2 receptors and Ca^2+^-dependent pathways (Feng et al., 2020; Feng et al., 2017; Li et al., 2019).

Additionally, gut microbiota can influence the levels of DA, thereby affecting brain arousal and activity. Relevant studies have indicated that dysbiosis of gut microbiota leads to DA metabolic disorders (characterized by a decrease in homovanillic acid). Supplementation with probiotics has been shown to restore populations of *Bacteroides*, *Blautia*, *Dialister*, *F. prausnitzii*, and *Ruminococcus*, which are significantly positively correlated with homovanillic acid levels (Wang et al., 2020). Moreover, *E. faecalis* and *Enterococcus* faecium possess tyrosine hydroxylase and dopa decarboxylase activities, enabling them to convert tyrosine into L-DOPA. Further research has demonstrated that transplantation of *E. faecalis* and E. faecium increases the synthesis of dopa/dopamine within the intestines of PGF mice, facilitating the entry of dopa/dopamine from the gut into the bloodstream, thus elevating dopamine levels in the brain (Wang Y. et al., 2021) (Figure 1). This suggests that modulating the biosynthesis pathway of phenylalanine-tyrosine-dopa-dopamine in gut microbiota could potentially improve brain function. Furthermore, research has demonstrated that the gut microbiota’s SCFAs can directly penetrate the BBB to control the synthesis of 5-HT and DA, which in turn affects brain development (Wang et al., 2020). Thus, gut bacteria can influence the brain’s arousal capacities and functions by modulating the brain’s dopaminergic system through a variety of pathways.

### 3.3 Microbiota-vagus nerve pathway

Vagal nerve fibers, which receive a variety of signals from the gut and precisely transfer them to the brain, are abundant in the intestinal wall. For instance, the gut microbiota’s metabolism of Trp can trigger enteroendocrine cells’ (EECs’) Trpa1 signaling, which in turn triggers the gut’s vagal nerve system (Ye L. et al., 2021). Through the vagal nerve system, gut bacteria can alter the brain’s GABA receptor expression levels, reducing anxiety and depressive symptoms (Zou et al., 2024). Similar to this, fecal microbiota transplantation can trigger the gut’s vagal nerve system, which results in long-lasting alterations to the brainstem and hippocampus’s 5-HT and DA neurotransmission pathways (Siopi et al., 2023). Through a vagus nerve-dependent mechanism, *Lactobacillus reuteri* can also alter how oxytocinergic and dopaminergic signals are transmitted in the ventral tegmental area (VTA) (Sgritta et al., 2019). Notably, both dopaminergic circuits in the hippocampus and the VTA play crucial roles in maintaining sleep-wake-related behaviors (Bian W. et al., 2022).

To confirm the involvement of the vagus nerve in information transmission between the gut and the brain, researchers conducted subdiaphragmatic vagotomy experiments. They found that this not only abolishes the therapeutic effects of selective 5-HT reuptake inhibitors but also significantly reduces the activity of nerve fibers showing immunoreactivity to the 5-HT3 receptor in the intestinal mucosa (Glatzle et al., 2002; McVey Neufeld et al., 2019). When 5-HT within the gut binds to densely distributed 5-HT receptors on vagal nerve fibers, the vagus nerve is activated. Neurons then respond to and categorize the 5-HT signals through specific projections before conveying them to the brain (Spencer et al., 2024) (Figure 1).

Research has demonstrated that vagus nerve stimulation can ameliorate depression-like behaviors brought on by SD by lowering the levels of interleukin-1β (IL-1β) and interleukin-6 (IL-6) in peripheral blood and the hippocampus, as well as by preventing astrocyte and microglia activation (Ma et al., 2024). Vagotomy not only alleviates the systemic inflammatory response caused by SD-induced gut microbiota dysbiosis but also weakens the effects of probiotics on neuropsychiatric disorders by reducing the signaling of microbial metabolites along the gut-brain axis (Yun et al., 2020; Zhang et al., 2021). As a result, the vagus nerve allows a variety of gut signals to reach the brain and influence many brain activities.

### 3.4 Microbe-immune inflammatory pathway

Microbes establish complex interactions with the brain’s sleep regulatory functions through immune-inflammatory pathways (Sgro et al., 2022). SD disrupts the gut barrier and BBB permeability, increases NLRP3 levels, and activates the TLR4/NF-κB signaling pathway in the gut, leading to the transmission of inflammatory signals to the brain, thereby exacerbating neuroinflammation and microglial activation in the brain (J. Sun et al., 2020; Wang Z. et al., 2021; Zhao N. et al., 2023). Restoring disrupted gut microbiota can alleviate gut oxidative stress, inflammatory responses, and barrier damage, thereby improving sleep quality (Lai W. D. et al., 2022; Li L. et al., 2023; Wu Z. et al., 2023). Certain gut microbes can regulate the brain’s sleep functions by mediating the host’s inflammatory response through their metabolites (Chen H. et al., 2023; Yin et al., 2024). For example, trimethylamine N-oxide (TMAO), a metabolite of gut microbiota, can enhance BBB integrity by modulating annexin A1 signaling, protecting the brain from inflammatory damage (Hoyles et al., 2021), and can also affect sleep by promoting NLRP3 activation through the NF-κB signaling pathway (Praveenraj et al., 2022) (Figure 1).

SD triggers gut microbiota dysbiosis, leading to an imbalance in the expression of inflammatory factors such as IL-1β, IL-6, and tumor necrosis factor α (TNF-α) (Zhang M. et al., 2023). Related analyses indicate that IL-1β and TNF-α are positively correlated with *Ruminococcus_1* and *Ruminococcaceae_UCG-005* in the gut (Yao et al., 2022). NLRP3 is one of the key mediators involved in IL-1β-controlled sleep regulation. Assembly of NLRP3 can activate caspase-1, and activated caspase-1 subsequently cleaves pro-IL-1β into mature IL-1β, which then regulates the CNS control of physiological sleep (Aghelan et al., 2023). TNF-α and IL-1β receptors mediate NF-κB transcription through ligand activation, triggering the transcription of inflammation-related molecules involved in sleep regulation and affecting sleep (Misrani et al., 2024). Although the impact of SD on the BBB is somewhat reversible, restoring normal BBB function remains a lengthy process even after resuming a regular sleep pattern (Axelrod et al., 2023; Puech et al., 2023).

## 4 Ethnopharmacology

### 4.1 Overview of traditional therapies

Traditional medicine, particularly traditional Chinese medicine (TCM), has long been used to treat insomnia. According to TCM theory, the onset of insomnia can be attributed to a number of factors, including mental health conditions, dietary choices, excessive work, physical exhaustion, and post-illness debility. These elements can result in a variety of clinical changes, such as liver yang hyperactivity, heart and spleen deficits, and yin deficiency with fire hyperactivity (Qin and Xiao, 2024). Therefore, individualized care and symptom distinction are highly valued in TCM approaches to insomnia treatment. Because treatment strategies are tailored to each patient’s specific etiology and pathophysiology, this ensures individualized therapy.

Chinese herbal medicine formulations are basic therapeutic units within the context of TCM, where various mixtures of medicinal herbs are customized to target particular disease causes. In China, many traditional remedies for insomnia have been preserved and are still in use today. For example, Suanzaoren Decoction is mentioned in the classic book Essential Prescriptions of the Jingui Yaolue (金匮要略), which was put together by the famous doctor Zhang Zhongjing (张仲景) (Rong et al., 2022). Again, one of the oldest pharmacopeias in the world, Taiping Huimin Heji Jufang (太平惠民和剂局方), mentions Guipi Decoction (Zhou, 2016).

Suanzaoren Decoction is composed of five medicinal herbs in the following amounts: 15 g of Ziziphi Spinosae Semen (Suanzaoren, SZR), 9 g of Poria cocos, 9 g of Anemarrhena asphodeloides Bunge, 6 g of Ligusticum chuanxiong, and 3 g of Glycyrrhiza uralensis Fisch. For conditions like restless insomnia, vivid nightmares, palpitations with night sweats, dizziness, and blurred vision caused by hepatic blood insufficiency and rising virtual heat, the major ingredient in this decoction is advised (Kui et al., 2022).

GuiPi Decoction is composed of ten medicinal herbs in the following proportions: 6 g of *Panax Ginseng*, 3 g of *Atractylodes macrocephala*, 3 g of *Angelica sinensis*, 3 g of *Poria cocos*, 3 g of *Astragalus mongholicus Bunge*, 3 g of *Polygala tenuifolia Willd*, 3 g of *D. longan Lour* (*D*. *longan Lour*), 3 g of SZR, 1.5 g of *Aucklandia lappa*, and 1 g of Processed *Glycyrrhiza uralensis Fisch*. This decoction is primarily used for its benefits in improving memory, calming palpitations, alleviating irritability and insomnia, and addressing spontaneous sweating and palpitations (Zhou, 2016).

Traditional herbal formulas comprise a diverse array of Chinese medicinal herbs, intended to address the various pathophysiological mechanisms underlying insomnia through comprehensive regulation, thereby achieving synergistic effects across multiple targets. However, the complexity of these formulas, which arises from the inclusion of multiple herbal components, can make it difficult to identify specific active ingredients and their precise mechanisms of action. Additionally, for acute or severe cases of insomnia, Chinese herbal medicine formulas may have a relatively slower onset of action, potentially requiring an extended period before significant therapeutic effects become apparent.

### 4.2 Trends in modern therapeutic approaches

Promoting TCM internationally is fraught with difficulties as modern medicine develops. The effective components of single herbs have been extensively studied through modern pharmacology, leading to clearer mechanisms of action and easier standardization of production processes, which ensures consistent and stable product quality. Nevertheless, excessive use of some single herbs or their extracts, especially those that include alkaloids or other strong substances, may result in negative side effects or dependence (Rice and Genest, 1965).

Insomnia can be effectively treated using medicinal Chinese herbs that have dual uses as food and medicine. These herbal medicines are appropriate for inclusion in regular diets in addition to their therapeutic benefits (Xin-yuan et al., 2025). They promote long-term conditioning and prevention by efficiently enhancing sleep quality without placing extra strain on the body (Shan et al., 2015). Additionally, the choice of therapeutic foods can be customized to fit specific needs, guaranteeing maximum effectiveness. For instance, in cases where insomnia is attributed to heart-spleen deficiency, herbs such as *D. longan Lour*. and *Ziziphus jujuba Mill* are recommended for their blood-nourishing and calming properties (Jing et al., 2023). For individuals experiencing insomnia due to yin deficiency and excessive fire, herbs like *Lilium lancifolium Thunb*. and lotus (*Nelumbo nucifera Gaertn.*) seeds are preferred for their ability to nourish yin and reduce heat (Shi et al., 2023).

## 5 Herbal medicines and their active components improve sleep quality by modulating gut microbiota

Medicinal foods derived from medicinal Chinese herbs, along with their bioactive compounds, serve as distinctive “prebiotics” that offer several advantages: minimal adverse effects, high safety profiles, enhanced patient compliance, and suitability for long-term consumption (Delzenne and Bindels, 2015). These properties highlight the dual role of these herbs in both nourishment and therapeutic applications, underscoring their unique value. Moreover, they possess the potential to regulate the stability of the gut microbiota, providing a novel perspective on the prevention and treatment of insomnia through modulation of the intestinal microbiome.

### 5.1 Ziziphi spinosae semen

Ziziphi spinosae semen (Suanzaoren, SZR) refers to the dried mature seeds of *Ziziphus jujuba Mill. var. spinosa (Bunge) Hu ex H. F. Chou*, a species within the Rhamnaceae family (Commission, 2020). Characterized by its sweet and sour taste with neutral properties, SZR targets the liver, gallbladder, and heart meridians. It is renowned for its functions of “nourishing heart yin, enriching liver blood, calming the mind, and stabilizing the spirit” (Tong et al., 2024), making it particularly effective for treating deficiency-type and chronic insomnia. Historically, SZR has been highly regarded in TCM. Due to its efficacy, both as a single herb and in compound formulations, SZR is widely used in clinical settings for the treatment of insomnia, earning it the accolade of “Eastern Sleep Fruit” (Yong-qing et al., 2024) (Table 1).

**TABLE 1:** By altering the microbiota-gut-brain axis, several Chinese herbal medicines and their active ingredients may enhance the quality of sleep.

Herbal name	Active components and administered parts	Effects	Animal model	Experimental group	Control group	Mechanism of action	Involved microbiota
Ziziphi spinosae semen	Aqueous extract	Restoring the sleep wake circadian rhythm of circadian rhythm sleep-wake disorder mice, improving sleep quality	Male Sprague Dawley rats, p-chlorophenylalanine (PCPA) (400 mg/kg, i.p. 3 days)	Aqueous extract (8.0 g/kg, oral, 5 days)	Diazepam group (0.92 mg/kg, oral, 5 days)	Further affect the amino acid metabolism pathway in the body by regulating the abundance of *Clostridium*, Lactobacilus and their metabolite butyric acid content	*Clostridium*, *Lactobacillus* and *Roseburia*, *Bifidobacterium*, *Ruminococcus*, *Eubacterium* and *Prevotella* (Du et al., 2022; Ling et al., 2024; Wang C. H. et al., 2023)
*Lilium lancifolium Thunb*	Aqueous extract	Improving Sleep and Alleviating Depression	Wistar rats, PCPA-induced sleep disturbance (400 mg/kg, i.p. 2 days)	Aqueous extract (598.64 mg/kg, oral, 7 days)	control group (saline, oral, 7 days), PCPA group (400 mg/kg, i.p. 2 days)	Reversing the adverse effects of insomnia on gut Microbiota diversity and richness control the metabolism of arachidonic acid and Trp within the gut and positively regulate 5-hydroxy-L-tryptophan in the hypothalamus, involved in the metabolism of 5-HT.	*Porphyromonadaceae, Lactobacillus, and Escherichia* (Si et al., 2022a; Si et al., 2022b)
*Ganoderma lucidum*	Aqueous extract, ethanolic extract, polysaccharide	Romotes sleep, induces sedation, alleviates anxiety, and provides neuroprotection	Male ICR mice	Ethanolic extract (25 mg/kg, 50 mg/kg, and 100 mg/kg, oral, 28 days)	control group (0.05% CMC-Na, oral, 28 days).	It increased the 5-HT levels in the brain and enhanced the production of important transcription factors, including Tph2, Iptr3, and Gng13	*Bacteroidetes, Actinobacteria, Bifidobacterium, Lactobacillus and Klebsiella* (Chen T. et al., 2022; Yao et al., 2021)
*Poria cocos*	Polysaccharide	Sedative-hypnotic, anxiolytic	Male Wistar rats, chronic sleep deprivation	water-soluble polysaccharide (100 mg/kg, oral, 21 days)	Model group (saline, oral, 21 days), Estazolam (0.18 mg/kg, oral, 21 days)	Upregulate the levels of key neurotransmitters 5-HT, DA, NE, and GABA in the hypothalamus, increase the number of neuronal cells, and simultaneously reduce the expression levels of inflammatory factors; enhance the gut barrier by increasing the levels of SCFAs in the gut and promoting Trp metabolism	*Firmicutes, Bacilli, Lactobacillales*, *Prevotellaceae*_UCG-001, and *Fusicatenibacter* (He et al., 2023; Zhang and Ye, 2022)
*Panax ginseng*	Ginsenoside Rb1, Rg1, Rg5	Neuroprotection, improvement of sleep quality	Male Sprague Dawley rats (by housing rats in a sleep deprivation box for 4 weeks)	Rg5 25 mg/kg Rg5 or 50 mg/kg Rg5	MT group (0.27 g/kg)	It corrects imbalanced gut microbiota and restores the function of the gut barrier and controls the metabolism of fat and glucose, raising the GABA/Glu ratio and upregulating the expression of GABAA, GABAB, and 5-HT receptor 1A	*Lactobacillus*, *Bacteroidetes* (Chen et al., 2020; Chen J. et al., 2023; Shao et al., 2020; Wei et al., 2020)
*Gastrodia elata Blume*	Gastrodin	Provides neuroprotection, and has antidepressant effects	ApoE^−/−^ mice, chronic mild stress-induced depressive	*Gastrodia elata* Blume water extract (10 mL/kg, 20 mL/kg, oral, 28 days)	fluoxetine group (20 mg/kg, oral, 28 days)	Reshape the gut microbiome structure by promoting the increase and balance of potentially beneficial bacteria and fecal SCFAs levels, normalizing the ratio of 5-hydroxyindoleacetic acid to 5-HT in the colon, and reducing the ratio of kynurenine to tryptophan	*Alloprevotella*, *Defluviitaleaceae* UCG-011, *Bifidobacterium*, *Akkermansia*, and *Parabifidobacter* (Huang et al., 2023; Huang et al., 2021)
*Astragalus mongholicus Bunge*	Polysaccharide	Improves sleep quality	Male C57BL/6 mice (by housing mice in a sleep deprivation box for 3 days)	Polysaccharide distilled water solution (0.5 g/L, 1.0 g/L, and 1.5 g/L, oral, 28 days)	sleep disturbance group (distilled water, oral, 28 days)	Ameliorate immunological dysfunction by regulating certain microorganisms implicated in inflammatory and immune responses, SCFAs synthesis, and the TLR4/NF-κB pathway, and raise the amounts of SCFAs and GABA in feces	*Pseudoflavonifractor*, *Paraprevotella*, *Oscillibacter*, *Tyzzerealla*, *Lachnoclostridium, Lactobacillus*, *Bifidobacterium*, *Roseburia*, *Desulfovibrio*, *Paracoccus*, *Parabacteroides*, *Clostridium XIVb*, and *Butyricicoccus* (Li et al., 2014; Li Y. et al., 2023; Zhao W. et al., 2023)
*Dimocarpus longan Lour*	polysaccharide	Improves sleep quality	—	—	—	boost the levels of succinic acid and SCFAs (acetate, propionate, and butyrate) and enhance gut immunity	*lactobacilli, pediococci,* and *bifidobacteria* (Bai et al., 2023; Zhang et al., 2017)
*Gardenia jasminoides J. Ellis*	Fruit, geniposide A	Improves sleep and anxiety	Male Sprague Dawley rats, PCPA-induced sleep disturbance (400 mg/kg, i.p. 3 days)	Fructus *gardenia* (1.05 g/kg, 4.15 g/kg, oral, 7 days)	Estazolam group (0.5 mg/kg, oral, 9 days)	Modulates hippocampal metabolites, reduces TNF-α and IL-7β levels, and regulates gut microbiota	Increases abundance of *Muribaculaceae* and *Lactobacillus*, decreases abundance of *Lachnospiraceae_NK4A136_group* (Alonso-Castro et al., 2020; Liu D. et al., 2023)
*Panax notoginseng* (Burkill) F. H. Chen	Notoginsenosides	Improves insomnia, depression, and cognitive impairment caused by SD	Male C57BL/6 mice, SD 2 days	Notoginsenosides groups (25 mg/kg, 50 mg/kg, and 100 mg/kg, oral, 9 days)	modafinil groups (13 mg/kg, oral, 9 days), SD groups (distilled water)	Inhibits abnormal autophagy and apoptosis of hippocampal neurons, regulates monoamine neurotransmitters and intracellular Ca^2+^ concentration in the brain	*Faecalibacteriumprausnitzi* (Cao Y. et al., 2020; Li et al., 2018; Shao et al., 2023)
Lotus *(Nelumbo nucifera Gaertn.)* seeds	Water extract of lotus seeds	Improves sleep	Male ICR mice, caffeine-induced sleep disturbance (10 mg/kg, oral, 4 days)	*Nelumbo nucifera* seeds water extract (80 mg/kg, 160 mg/kg, oral, 9 days)	BDZ group (2.5 mg/kg, oral, 9 days) Caffeine (10 mg/kg, oral, 4 days)	Regulates GABA receptors	LOS3-4 and LOS1 increase the abundance of *Lactobacillus* acidophilus, LOS4 increases the abundance of *Bacteroides* and *Bifidobacterium* (Joet al., 2021; Lei et al., 2022; Ma et al., 2023)
Lotus (*Nelumbo nucifera Gaertn.*) leaves	Total alkaloids, quercetin-3-O-glucuronide	Sedative and hypnotic	Male Sprague Dawley rats, caffeine-induced sleep disturbance (80 mg/kg, oral)	Ethanol Extract of Lotus Leaf (150 mg/kg, 300 mg/kg, oral, 9 days)	caffeine group (80 mg/kg, oral, 9 days)	Alkaloids bind to GABAA receptors and activate monoaminergic systems; quercetin-3-O-glucuronide regulates GABA pathway	Quercetin-3-O-glucuronide increases the abundance of *Actinobacteria* and *Firmicutes*, decreases the abundance of *Proteobacteria* (Feng et al., 2023; Kim et al., 2021; Yan et al., 2015)
*Camellia sinensis* (L.) Kuntze	L-Theanine, caffeine, tea polyphenols, tea pigments, tea polysaccharides	Improves sleep	—	—	—	cycles via neuroendocrine pathways and immune system; promotes Trp metabolism and 5-HT pathway to regulate circadian rhythms	Lowers F/B ratio, increases the abundance of *Bacteroides* and *Prevotella* (Guo et al., 2019; Hu et al., 2022; Li et al., 2023; Wei et al., 2023)
*Setaria italica (L.) Beauv*	Probiotic fermentation products of germinated grains	Improves sleep	Female C57BL/6 mice, PCPA (0.4 mg/10 g, i.p. 2 days)	Experimental (with an average daily intake of 30–35 mL per group, oral, 14 days)	Diazepam group (0.025 mg/mL, oral, 14 days) Model (free water, oral, 14 days)	Regulates neurotransmitter and inflammatory factor levels, increases SCFAs content	Increases the abundance of *Prevotella*, *Lactobacilli*, and *Ruminococcus*, decreases the abundance of *Muribaculaceae* and *Erysipelotrichaceae* (Cheng J. et al., 2024)
*Ziziphus jujuba Mill*	Extract and polysaccharides of jujube	Improves sleep, neuroprotective effects	Male Sprague Dawley rats, caffeine-induced sleep disturbance (80 mg/kg, oral, 9 days)	The water extract of jujube seeds (100 mg/kg, 150 mg/kg, oral, 9 days)	BDZ group (0.2 mg/kg, oral, 9 days)	Regulates GABAergic system	Upregulates the abundance of *Lachnoclostridium* and *Marvinbryantia*, downregulates the abundance of *Alistipes* and *Akkermansi*a (Bae et al., 2023; Li Z. et al., 2023)
*Lycium barbarum L*	Polysaccharides, anthocyanins, and alkaloids	Anti-fatigue, neuroprotective effects	—	—	—	Anti-fatigue: Anthocyanins regulate ERK/MAPK signaling pathway and reduce CRF release; Neuroprotective: Anthocyanins improve gut barrier, inhibit TLR4 signaling pathway	Anthocyanins: Increase the abundance of *Lactobacilli*; Lycium polysaccharides: Increase the abundance of *Lactobacillus* and *Bifidobacterium* (Dong et al., 2023; Peng et al., 2023; Tian et al., 2024)

Saponins, flavonoids, and alkaloids are the main active components responsible for the sedative and hypnotic effects of SZR (Bian Z. et al., 2022). Previous studies have shown that SZR can regulate physiological processes such as amino acid metabolism, neurotransmitter release, inflammatory factor balance, and HPA axis homeostasis, thereby modulating neural activity and achieving equilibrium in the nervous system to improve sleep (Dong et al., 2021; Liu et al., 2022; Xiao F. et al., 2022).

Recent studies have indicated that SZR can restore the imbalance of gut microbiota caused by insomnia, increasing the relative abundance of beneficial bacteria such as *Lactobacillus*, *Bifidobacterium*, *Lactococcus*, and *Eubacterium* in rats, while simultaneously reducing the relative abundance of *Prevotella* (Du et al., 2022; Wang C. H. et al., 2023). The increased abundance of *Clostridium* and *Lactobacillus* leads to an increase in the content of their metabolic product, butyrate, which further affects amino acid metabolism pathways in the body, thereby significantly restoring the sleep-wake rhythm in mice with sleep-wake rhythm disorders (Ling et al., 2024). SCFAs can indirectly regulate the levels of 5-HT in both the gut and the brain (Mukherjee et al., 2020; Yu et al., 2020). Moreover, research has shown that the SCFAs increased through microbial metabolism of sour SZR can modulate the expression of representative factors in the TLR4/NF-κB/NLRP3-related signaling pathway in the colon, thereby regulating the microbiota-gut-brain axis and improving insomnia and depressive behaviors in mice (Du et al., 2023; Hua et al., 2021).

**FIGURE 2:**
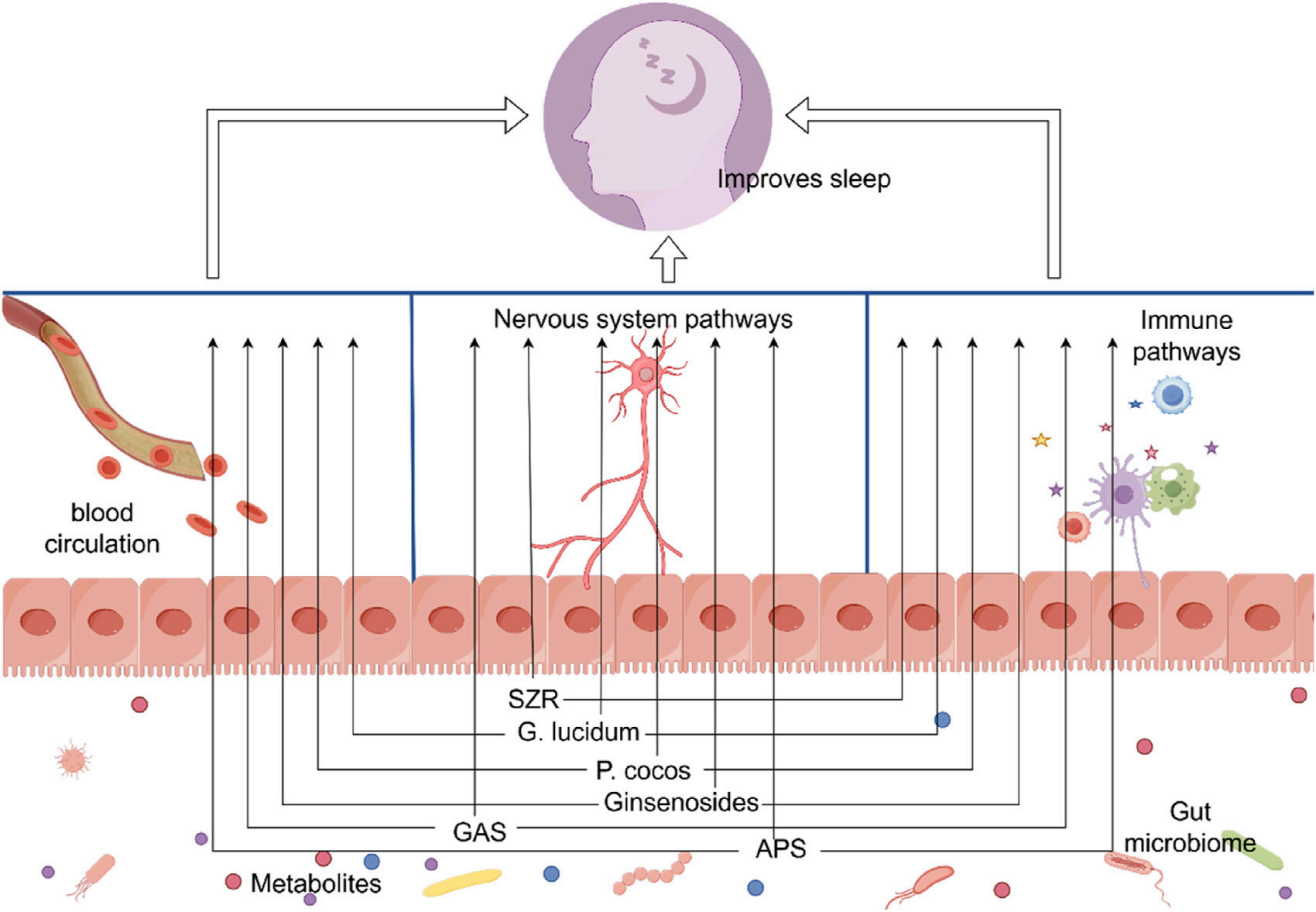
Through altering the gut microbiome, Chinese herbal remedies and their active ingredients can enhance sleep. SZR, ziziphi spinosae semen; *G. lucidum*, *Ganoderma lucidum*; *P. cocos*, *Poria cocos*; GAS, Gastrodin; APS, Astragalus polysaccharide. Materials provided by FigDraw (www.fgdraw.com).

It is evident that SZR can regulate the microbiota-gut-brain axis through both the microbiota-neural pathway and the microbiota-immune pathway, thereby achieving control over the organism’s sleep behavior (Figure 2). This may explain how the active components of SZR, which cannot cross the BBB, still manage to improve sleep quality.

### 5.2 Lilium lancifolium Thunb

*Lilium lancifolium Thunb* (LB) can improve hypothalamic pathology and alleviate insomnia behavior in rats by reducing the levels of HPA axis hormones in the serum, increasing the levels of 5-HT and MT, decreasing the levels of noradrenaline (NE) in the hypothalamus, and upregulating the expression of GABAA receptors and 5-HT1A receptors (Si et al., 2020).

The way that LB regulates the gut flora may have a direct bearing on how well it relieves sleeplessness. In addition to reversing the detrimental effects of insomnia on the diversity, abundance, and fecal metabolic phenotype of the gut microbiota, LB also controls the metabolism of arachidonic acid and Trp, which lowers the expression levels of kynurenine, a chemical linked to mental health issues (Si et al., 2022b). LB significantly modulates the relative abundances of *Porphyromonadaceae*, *Lactobacillus*, and *Escherichia* within the gut and positively regulates 5-hydroxy-L-tryptophan in the hypothalamus, which is involved in the metabolism of 5-HT. With LB intervention, the most important and fundamental pathways are thought to be the serotonergic system and the arachidonic acid metabolic pathway (Si et al., 2022a) (Table 1). Additionally, the decoction of LB and *Rehmannia glutinosa (Gaertn.)* (LBRD) can lessen neuronal damage linked to depressed behavior by rectifying gut microbiota dysbiosis and avoiding inflammation brought on by compromised gut barriers or BBB leaking. Through the microbiota-gut-brain axis, *Lactobacillus*, *Bifidobacterium*, and *Lactococcus* are shown to be important contributors to the antidepressant effects of LBRD, according to a fecal microbiota transplantation and gut microbiota composition study (Mao et al., 2024).

### 5.3 Ganoderma lucidum

Research has found that the calming effects of *Ganoderma lucidum* (*G. lucidum*) are closely linked to the gut microbiota. When *G. lucidum* is fermented with *Lactobacillus reuteri*, it can increase the levels of 5-HT and GABA in the serum and reduce the levels of HPA axis hormones, effectively alleviating insomnia symptoms in anxious mice (Huang et al., 2022). The ethanol extract of *G. lucidum* also demonstrates a significant impact on the structure of the microbiome. At the phylum level, it increases the abundance of *Bacteroidetes* and *Actinobacteria*; at the genus level, it increases the abundance of *Bifidobacterium* while decreasing the abundance of *Lactobacillus* and *Klebsiella* (Chen T. et al., 2022). The hypothalamus’s 5-HT concentrations are positively correlated with *Bifidobacterium* and *Bifidobacterium animalis*, which not only raises 5-HT levels there but also increases the production of important transcription factors including Tph2, Iptr3, and Gng13 (Yao et al., 2021). After depleting the gut microbiota with antibiotics, the sleep-promoting effects of the ethanol extract of *G. lucidum* and the changes in fecal metabolites associated with sleep behavior completely disappear (Yao et al., 2021). This indicates that the gut microbiota plays an indispensable role in the sleep-promoting effects of *G. lucidum* (Table 1).

Furthermore, polysaccharides derived from *G. lucidum* have been shown to enhance GABA and 5-HT levels in mice’s brains and enhance the quality of their sleep by lengthening delta waves during NREMS (Ye H. et al., 2021). In addition, polysaccharides from *G. lucidum* and their hydrolysis products, known as ganoderan peptides, exhibit immunomodulatory activities through various pathways. Specifically, *G. lucidum* polysaccharides not only reduce the F/B ratio in the gut but also significantly upregulate the expression levels of NF-κBp65, IL-2, and IL-4 in the ileum (Jin et al., 2017); they also significantly enhance the expression of BDNF, inhibit the expression of pro-inflammatory cytokines, and suppress the activation of microglia and astrocytes, thereby exerting an antidepressant effect (Li et al., 2021) (Figure 2). Ganoderan peptides are involved in key pathways such as the folate cycle, biosynthesis and metabolism of fatty acids, and cAMP metabolism (Xie et al., 2023). *G. lucidum* polysaccharides have significant potential in regulating gut microbiota homeostasis and providing neuroprotection.

### 5.4 Poria cocos

As one of the preferred core medicines used by ancient people to treat insomnia, *Poria cocos* (*P. cocos*) contains polysaccharides and triterpenoids, which are its main active ingredients for calming the mind and inducing tranquility (Lai Y. et al., 2022; Li et al., 2022). Studies have shown that acidic polysaccharides from *P. cocos* can exert a calming effect by intervening in the gut microbiota and regulating related pathways. These polysaccharides not only increase the abundance of gut microbiota such as *Firmicutes*, *Bacilli*, *Lactobacillales*, *Prevotellaceae_UCG-001*, and *Fusicatenibacter*; they also upregulate the levels of key neurotransmitters 5-HT, DA, NE, and GABA in the hypothalamus, increase the number of neuronal cells, and simultaneously reduce the expression levels of inflammatory factors (Zhang and Ye, 2022). Additionally, aqueous extracts and water-soluble polysaccharides from *P. cocos* can prevent the onset of anxiety effectively by improving gut microbiota imbalances, alleviating metabolic disorders, regulating the levels of gut neuropeptides and neurotransmitters, and inhibiting the TNF-α/NF-κB signaling pathway (Zhang D. et al., 2022; Zhang D. D. et al., 2022) (Figure 2).

Furthermore, polysaccharides from *P. cocos* can enhance the gut barrier by increasing the levels of SCFAs in the gut and promoting Trp metabolism. They not only promote Trp metabolism by increasing the levels of indole lactate and indole-3-aldehyde (He et al., 2023) (Table 1); they also maintain the integrity and function of the gut barrier by increasing the levels of SCFAs in the gut, regulating the expression of key immune factors such as IL-2, IL-4, IL-6, IL-10, TGF-β, and IFN-γ, and activating the Wnt/β-Catenin signaling pathway (Duan Y. et al., 2023). The levels of Trp and SCFAs, as well as the healthy homeostasis of the gut barrier, are closely linked to the gut microbiota and have significant impacts on the improvement of sleep quality.

### 5.5 Panax ginseng

Peptides, polysaccharides, and saponins found in *Panax ginseng* have the ability to cross the blood-brain barrier and regulate the neuroendocrine balance and metabolic environment of the brain (LU et al., 2023). *Panax ginseng* polysaccharides enhance ginsenoside Rb1 and microbial metabolite exposure by enhancing intestinal absorption and affecting gut microbial metabolism (Shen et al., 2018). Rb1 exerts neuroprotective effects through regulation of *Lactobacillus* helveticus abundance and GABAA receptor expression (Chen et al., 2020). Ginsenoside Rg1 (Rg1) has potential health-promoting effects on the nervous system. Rg1 can ameliorate morphine-induced gut microbiota dysbiosis (specifically for Bacteroidetes), inhibit gut microbiota-derived tryptophan metabolism, and reduce serotonin, 5-hydroxytryptamine receptor 1B, and 5-hydroxytryptamine receptor 2A levels (Han et al., 2020).

*Ginsenoside Rg* may affect the gut microbiota and offer neuroprotection through a number of different pathways. Rg1 can keep mice from developing a morphine dependence by regulating the 5-HT neurotransmitter system, preventing the metabolism of Trp produced by the gut microbiota, and improving gut microbiota dysbiosis (particularly with regard to Bacteroidetes) (Chen Z. et al., 2022) (Table 1). Rg5 not only corrects imbalanced gut microbiota and restores the function of the gut barrier, but it also alters the rhythmic characteristics of clock-related proteins and improves the quality of your sleep by controlling the metabolism of fat and glucose, raising the GABA/Glu ratio, and upregulating the expression of GABAA, GABAB, and 5-HT receptor 1A, which in turn affects the GABA and 5-HT neurotransmitter systems (Chen J. B. et al., 2023; Shao et al., 2020; Wei et al., 2020).

Furthermore, the combined use of *P. ginseng* and SZR can improve the structure of the gut microbiota, promote the proper functioning of the Glu/GABA-Gln metabolic cycle, and increase the synthesis and release of GABA in the hippocampus, thereby significantly improving the sleep state in insomniac rats (Qiao et al., 2022). This suggests that *P. ginseng* can intervene in the gut microbiota and modulate brain sleep functions through multiple pathways (Figure 2).

### 5.6 Gastrodia elata Blume

The extraction and isolation of gastrodin (GAS) from *Gastrodia elata Blume* (*G*. *Blume*) have been shown to have a notable impact on illnesses of the central nervous system, including but not limited to sleeplessness, anxiety, depression, cognitive impairment, and ischemic stroke (Zhang et al., 2020). By controlling the expression of inflammatory factors like IL-6 and IL-1β, the activity of B-cell lymphoma-2 (Bcl-2) protein, and the ratio of p-ERK to ERK, GAS not only improves the quality of sleep in mice with insomnia (Long et al., 2021), but it also ameliorates sleep disorders brought on by the deprivation of rapid eye movement sleep (REMS) by modulating the TLR4/NF-κB and Wnt/β-catenin signaling pathways (Liu B. et al., 2023). GAS, therefore, has a lot of potential to enhance the quality of sleep.

Studies already conducted show that GAS can inhibit neuronal apoptosis by regulating the microbiota-gut-brain axis, which improves cognitive impairment and neurodegeneration in AD mice (Zhang Y. et al., 2023). In depressed mice, the aqueous extract of *G. elata Blume* can reshape the gut microbiome structure by promoting the increase and balance of potentially beneficial bacteria (*Alloprevotella*, *Defluviitaleaceae UCG-011*, *Bifidobacterium*, *Akkermansia*, and *Parabifidobacter*) and fecal SCFAs levels, normalizing the ratio of 5-hydroxyindoleacetic acid to 5-HT in the colon, and reducing the ratio of kynurenine to tryptophan (Huang et al., 2023; Huang et al., 2021) (Table 1). The administration of a mixture of antibiotics partially abolishes the neuroprotective effects of GAS in AD mice (Fasina et al., 2022). However, conclusive experimental evidence supporting the notion that GAS can modulate the brain’s sleep function through this microbiota-gut-brain axis remains to be seen.

The effects of *G*. *Blume* and its active components on brain function are evident, as they partially target the microbiota-gut-brain axis. Further research is needed to determine the exact processes by which GAS improves sleep quality by altering the gut microbiota in order to enable the more prudent development and usage of *G*. *Blume* (Figure 2).

### 5.7 Astragalus mongholicus Bunge

Rich in flavonoids, polysaccharides, and saponins, *Astragalus mongholicus Bunge* helps to keep the gut microenvironment stable by controlling the composition, metabolism, and activity of the gut microbiota (Su et al., 2023).

Studies have found that different concentrations of *Astragalus Polysaccharides* (APS) have a mitigating and protective effect on the spleen and bodily injuries in SD mice (Li et al., 2014). Further research indicates APS can ameliorate immunological dysfunction by regulating certain microorganisms implicated in inflammatory and immune responses, SCFAs synthesis, and the TLR4/NF-κB pathway. For instance, APS can improve the immune function of immunocompromised mice and rats due to decreasing the abundance of *Pseudoflavonifractor*, *Paraprevotella*, *Oscillibacter*, *Tyzzerealla*, and *Lachnoclostridium* and increasing the abundance of *Lactobacillus*, *Bifidobacterium*, *Roseburia*, *Desulfovibrio*, *Paracoccus*, *Parabacteroides*, *Clostridium XIVb*, and *Butyricicoccus* (Li Y. et al., 2023; Zhao W. et al., 2023). However, in immunocompromised mice with reduced gut microbiota, APS did not improve immunological function (Li Y. et al., 2023) (Table 1, Figure 2).

In order to positively control the gut-brain axis, APS not only increases the relative abundance of *Lactobacillus* and *Bacillus* in the gut but also raises the amounts of SCFAs and GABA in feces and improves shrimp immunity (Sun et al., 2023). Additionally, APS can significantly attenuate age-associated disruption of the intestinal barrier, loss of gastrointestinal acid-base balance, reduction in intestinal length, overproliferation of the intestinal stem cells, and sleeping disorders upon aging (Li X. et al., 2023). Therefore, the gut microbiota not only actively aids in the body’s absorption of APS, but it also improves immunological dysfunction by raising gut SCFAs levels and positively regulates the gut-brain axis by encouraging the release of neurotransmitters.

### 5.8 Dimocarpus longan Lour

*Dimocarpus longan Lour*. (*D*. *longan Lour*.) flesh is one of the herbal remedies commonly used in traditional Chinese medicine to treat insomnia, but there is a dearth of pharmacological activity studies on its potential to improve the quality of sleep. *D*. *longan Lour*, which contains GABA, can be used as a natural dietary supplement. Numerous amino acids, such as GABA and Glu, are abundant in longans and are observed to increase in concentration as the fruit ages. Storage at refrigerated temperatures after maturation can increase the GABA content in the fruit (Hu et al., 2021; Zhou et al., 2016). Additionally, by increasing the amount of *lactobacilli*, *pediococci*, and *bifidobacteria* in the stomach, the gut microbiota’s metabolism of longan polysaccharides can boost the levels of succinic acid and SCFAs (acetate, propionate, and butyrate) and enhance gut immunity (Bai et al., 2023; Zhang et al., 2017) (Table 1).

It is evident that, whether as a dietary supplement rich in GABA or by modulating microbial metabolites within the gut, the flesh of *D*. *longan Lour* has the potential to regulate the microbiota-gut-brain axis by affecting the synthesis and release of sleep-related neurotransmitters such as GABA, MT, and 5-HT in the gut. This can directly or indirectly modulate the body’s sleep-wake cycle, thereby exerting a potent sleep-promoting effect.

### 5.9 Other Chinese herbal medicines and their components

Fewer studies have been conducted on herbs like *Gardenia jasminoides, Panax notoginseng*, and lotus seeds and their effects on gut microbiome interventions to improve sleep quality than on the aforementioned herbs and their active ingredients. In order to determine which herbs and which active ingredients enhance the quality of sleep, this article examines the pertinent literature. It also discusses how these active ingredients affect the gut flora. Through gut microbiome therapies, the ability of these herbs and their active ingredients to enhance sleep quality is assessed. As shown in Table 1.

Studies have shown that *Gardenia jasminoides J. Ellis* can improve sleep quality and alleviate anxiety symptoms by modulating the gut microbiota and reducing levels of TNF-α and IL-7β (Liu D. et al., 2023). *Panax notoginseng* saponins, metabolized by gut microbiota, are converted into ginsenoside Rg, which protects hippocampal neurons and regulates neurotransmitter levels in the brain, thereby improving insomnia, depression, and cognitive impairments (Cao Y. et al., 2020; Li et al., 2018; Shao et al., 2023).

Both lotus seeds and lotus leaves can regulate the GABAergic system by modulating the abundance and structure of the gut microbiota, thereby promoting sleep (Jo et al., 2021; Kim et al., 2021; Yan et al., 2015). However, there are significant differences in how they regulate the gut microbiota. Specifically, the oligosaccharide components in lotus seeds can increase the relative abundance of *Lactobacillus acidophilus*, *Bacteroides*, and *Bifidobacterium* (Lei et al., 2022; Ma et al., 2023). Lotus leaves contain flavonoid components that can increase the relative abundance of *Firmicutes* and *Actinobacteria* and reduce the abundance of *Proteobacteria* (Feng et al., 2023). *Ziziphus jujuba Mill* has the potential to improve sleep quality and provide neuroprotection through modulating the GABAergic system. Specifically, it has been shown to upregulate the abundance of *Lachnoclostridium* and *Marvinbryantia* while downregulating the abundance of *Alistipes* and *Akkermansia* (Bae et al., 2023; Li Z. et al., 2023). Additionally, *Setaria italica (L.) Beauv* may increase the abundance of *Prevotella*, *lactic acid bacteria*, and *Ruminococcus* in the gut, thereby increasing the levels of SCFAs and improving sleep quality by regulating neurotransmitter and inflammatory factor levels (Cheng J. et al., 2024). *Camellia sinensis (L.) Kuntze* may influence neuroendocrine pathways and the immune system by improving the gut microbiota, thereby regulating the sleep-wake cycle (Wei et al., 2023). *Lycium barbarum* may protect the nervous system by improving the gut barrier and inhibiting TLR4 signaling pathways (Dong et al., 2023; Tian et al., 2024).

The considerable potential of herbs and their active ingredients in modifying the gut microbiota and enhancing sleep quality has already been shown, despite the fact that this field of study is still in its early phases. Not only can more research provide solid support for treating insomnia, but it can also help us develop and use these herbs more sensibly by illuminating the mechanisms by which they interact with the microbiota-gut-brain axis to regulate brain functions related to sleep.

## 6 Conclusion and perspectives

This article focuses on the research of the microbiota-gut-brain axis, with an emphasis on exploring the pathways and targets for the prevention and treatment of insomnia through the intervention of gut microbiota. It also summarizes recent advances in the study of traditional Chinese herbal medicines and their active components in improving sleep quality by modulating the gut microbiota. Although the specific mechanisms by which gut microbiota participate in sleep regulation have not been fully elucidated, there is compelling evidence that gut microbiota and their metabolites can rapidly transmit relevant information to core brain regions via multiple pathways, including the nervous, endocrine, and immune systems, thereby modulating the brain’s sleep-wake cycle. Traditional Chinese herbal medicines and their active components have demonstrated significant potential in the prevention and treatment of insomnia, with a unique advantage lying in their effective modulation of the gut microbiota. By adjusting the composition of gut microbiota and their metabolites, these herbal medicines and their active ingredients can not only regulate multiple signaling pathways involving neurotransmitters and inflammatory mediators but also optimize physiological functions at several key targets, ultimately achieving improvements in sleep quality.

Despite significant progress in exploring the complex bidirectional interactions between the gut microbiota and insomnia, the intricate interplay between the gut microbiota and the brain, coupled with the multi-component and multi-target nature of Chinese herbal medicines, presents substantial challenges for further research in this field. (1) Model Limitations: Current studies on the relationship between gut microbiota and insomnia predominantly rely on germ-free (GF) or antibiotic-induced microbiota-depleted (AIMD) mouse models. However, these rodent models exhibit significant differences from humans in terms of sleep-wake cycle characteristics, potentially leading to biases when translating findings to human applications. To achieve a more precise and comprehensive understanding of the specific roles and mechanisms of gut microbiota in sleep health and disorders, it is essential to expand research into non-rodent or primate animal models and increase sample sizes to ensure the reliability of conclusions. (2) Complex Mechanisms of Action: The interaction between the gut microbiota and the brain involves multiple pathways, including neural, endocrine, and immune systems. The detailed mechanisms underlying these interactions remain incompletely understood, particularly regarding how Chinese herbal medicine components influence sleep through the “microbiota-gut-brain axis.” To address this, we should strengthen research on specific Chinese herbal medicine components, gut microbiota community structures, and their metabolic products, exploring how these factors jointly regulate brain function and sleep quality. This will deepen our understanding of the mechanisms of the “microbiota-gut-brain axis” and the therapeutic effects of Chinese herbal medicine interventions on this system. (3) Component Diversity and Target Identification: Chinese herbal medicines often contain multiple active components that may influence human health through various pathways. Identifying the key components responsible for improving insomnia and clarifying their exact targets within the microbiota-gut-brain axis is a challenging task. This requires fostering interdisciplinary collaboration among fields such as physiology, pharmacology, molecular biology, microbiology, and neuroscience. Integrating knowledge and technologies from these domains—such as leveraging bioinformatics for big data analysis to reveal complex biological networks—is essential. (4) Personalized Treatment Needs: Given the significant variability in gut microbiota composition among individuals, even the same Chinese herbal medicine herb or its components may exhibit different effects across different people. Therefore, when developing broadly effective treatments, it is crucial to account for this high degree of individual variability. A precision medicine approach—tailoring personalized treatment plans based on an individual’s microbiome profile—is a feasible strategy. However, this necessitates the development of advanced diagnostic tools and technologies to accurately analyze individual microbiota compositions and predict responses to specific herbs or components. (5) Standards and Quality Control: The extraction of active ingredients and formulation processes for Chinese herbal medicines are relatively complex, posing challenges in ensuring consistent product quality and stability. Establishing strict standards for Chinese herbal medicine ingredient extraction, formulation, and quality control systems is critical to ensuring the safety and efficacy of the products. Additionally, enhancing the identification and quantitative analysis of active Chinese herbal medicine components can improve drug consistency and reproducibility. (6) The Importance of Clinical Trials: While preliminary studies suggest that Chinese herbal medicines may improve sleep quality by modulating gut microbiota, these studies are often small-scale and lack rigorous design. The absence of large-scale, multicenter, randomized, double-blind, placebo-controlled clinical trials undermines the persuasiveness and credibility of Chinese herbal medicine efficacy claims. Furthermore, further research is needed on the long-term safety of Chinese herbal medicine use or its combination with other drugs, particularly when it involves modulating brain function via the gut microbiota. Potential adverse effects and side effects require special attention. In the future, rigorously designed large-scale clinical trials are necessary to validate the efficacy of Chinese herbal medicines and their active components in improving sleep through the modulation of the microbiota-gut-brain axis. Long-term follow-up studies should also be considered to assess the sustained effects on chronic insomnia patients and potential issues of dependency or drug resistance.

In the future, by implementing the measures outlined above and conducting multidimensional comparisons between Chinese herbal medicine herbs and existing sleep aids (such as melatonin and benzodiazepines) through high-quality randomized controlled trials, appropriate efficacy indicators, systematic collection and analysis of adverse reactions, exploration of mechanisms of action, and long-term follow-up studies, we can gain a more comprehensive understanding of the advantages and limitations of Chinese herbal medicines compared to conventional sleep aids. This will provide a solid scientific basis for their rational application.

## Glossary

CNS: Central nervous system
HPA: Hypothalamic-pituitary-adrenal
SD: Sleep deprivation
F/B: Firmicutes-to-Bacteroidetes
MUC2: Mucin 2
LPS: Lipopolysaccharides
SCFAs: Short-chain fatty acids
BBB: Blood-brain barrier
CORT: Corticosterone
CRF: Corticotropin-releasing factor
GABA: γ-Aminobutyric acid
MT: Melatonin
Trp: Tryptophan
5-HT: 5-Hydroxytryptamine
Kyn: Kynurenine
AANAT: N-acetyltransferase
Glu: Glutamate
Gln: Glutamine
NREM: Non-rapid eye movement
REMS: Rapid eye movement sleep
GI: Gastrointestinal
5-HTTP: 5-hydroxytryptophan
TPH: Tryptophan hydroxylase
TCM: Traditional Chinese medicine
EC: Enterochromaffin
EEC: Enteroendocrine
AADC: Aromatic L-amino acid decarboxylase
IL-33: Interleukin 33
SERT: Serotonin transporter
DA: Dopamine
VTA: Ventral tegmental area
TMAO: Trimethylamine N-oxide
TNF-α: Tumor necrosis factor α
NLRP3: NOD-like receptor thermoprotein domain protein 3
LB: *Lilium lancifolium Thunb*
LBRD: *Lilium lancifolium Thunb* and *Rehmannia glutinosa (Gaertn.) Libosch.*
GAS: Gastrodin
